# Scopolamine promotes neuroinflammation and delirium-like neuropsychiatric disorder in mice

**DOI:** 10.1038/s41598-021-87790-y

**Published:** 2021-04-16

**Authors:** So Yeong Cheon, Bon-Nyeo Koo, So Yeon Kim, Eun Hee Kam, Junhyun Nam, Eun Jung Kim

**Affiliations:** 1 Department of Biotechnology, College of Biomedical and Health Science, Konkuk University, Chungju, Republic of Korea; 2 Department of Anesthesiology and Pain Medicine, Yonsei University College of Medicine, 50-1 Yonsei-ro, Seodaemun-gu, Seoul, 03722 Republic of Korea; 3 Anesthesia and Pain Research Institute, Yonsei University College of Medicine, Seoul, Republic of Korea

**Keywords:** Neuroscience, Cognitive ageing, Cognitive neuroscience, Emotion, Learning and memory, Neuroimmunology, Immunology, Cell death and immune response, Cytokines, Inflammation, Neuroimmunology

## Abstract

Postoperative delirium is a common neuropsychiatric syndrome resulting a high postsurgical mortality rate and decline in postdischarge function. Extensive research has been performed on both human and animal delirium-like models due to their clinical significance, focusing on systematic inflammation and consequent neuroinflammation playing a key role in the pathogenesis of postoperative cognitive dysfunctions. Since animal models are widely utilized for pathophysiological study of neuropsychiatric disorders, this study aimed at examining the validity of the scopolamine-induced delirium-like mice model with respect to the neuroinflammatory hypothesis of delirium. Male C57BL/6 mice were treated with intraperitoneal scopolamine (2 mg/kg). Neurobehavioral tests were performed to evaluate the changes in cognitive functions, including learning and memory, and the level of anxiety after surgery or scopolamine treatment. The levels of pro-inflammatory cytokines (IL-1β, IL-18, and TNF-α) and inflammasome components (NLRP3, ASC, and caspase-1) in different brain regions were measured. Gene expression profiles were also examined using whole-genome RNA sequencing analyses to compare gene expression patterns of different mice models. Scopolamine treatment showed significant increase in the level of anxiety and impairments in memory and cognitive function associated with increased level of pro-inflammatory cytokines and NLRP3 inflammasome components. Genetic analysis confirmed the different expression patterns of genes involved in immune response and inflammation and those related with the development of the nervous system in both surgery and scopolamine-induced mice models. The scopolamine-induced delirium-like mice model successfully showed that analogous neuropsychiatric changes coincides with the neuroinflammatory hypothesis for pathogenesis of delirium.

## Introduction

Postoperative cognitive dysfunction (POCD) is a common complication of the central nervous system in the postoperative period with manifestations such as damage to memory, mental capacity, language ability, or other aspects of cerebral function^1^ and with an acute phase of cognitive impairment defined as postoperative delirium (POD)^2^. POD shows characteristic clinical features such as inattention, mood changes, and psychiatric disorders^3^. POCD and POD have been associated with clinically significant adverse outcomes, including prolonged hospitalization, decreased quality of life, and increased postoperative complications and mortality^4,5^. As the exact mechanism of POCD and POD remains to be explored, the activation of inflammation and immune systems is strongly regarded as the key mechanism for cognitive deterioration in postoperative period^6^.

Forebrain cholinergic neurons play a fundamental role in controlling the central nervous system with regards to attention, memory and cognitive function, and are implicated in cognitive decline and several neurodegenerative diseases^7–9^. The impact of cholinergic pathways on the immune system is also well-documented^7^ showing that systemic inflammatory responses are under the control of the cholinergic anti-inflammatory pathway supplied by connections of the vagus nerve^7,10^. Cholinergic signaling can also control peripheral cytokine production by cholinergic anti-inflammatory pathway activity^11^. It is reported that alterations in cholinergic system is involved in postoperative delirium in elderly patients^12^. In a similar context, experimental and clinical studies focusing on the pathogenesis for delirium show that it is accompanied by cholinergic pathways and agents^13,14^.

Scopolamine is an anti-cholinergic drug that antagonizes the muscarinic cholinergic receptors (mAChRs) and is capable of producing deficits in the processes of learning acquisition, and consolidation^15^. Scopolamine-treated animal models are widely used in neurocognitive studies because scopolamine administration induces both the behavioral and molecular features of Alzheimer’s disease and other neurocognitive disorders, including impaired cognition, and imbalanced cholinergic transmission in the hippocampus and prefrontal cortex^16–18^. In relation with the delirium occurrence, treatment with scopolamine had been conferred a relative risk for postoperative delirium in patients undergoing orthopedic surgery^19^. Scopolamine patch had also induced scopolamine-induced mental disorder especially in the elderly^20^.

Despite the highly analogous clinical traits and alterations in cholinergic neurotransmission in scopolamine-treated animal models^21^, the exact pathogenesis related to inflammatory response of POD still remains insufficient. The aim of this study was to validate the effectiveness of scopolamine-treated animal models as POD experimental model, by identifying the change in inflammation-related cytokines and inflammasome components in various brain regions and candidate genes using RNA sequencing technology by comparing a scopolamine-induced delirium-like mice model and surgery mice.

## Methods

### Animals and ethics statement

Male C57BL/6 mice aged 9–12 weeks (25–28 g) from Orient Bio (Seongnam, Gyeonggi-Do, South Korea) were used for the experiment. All in vivo experimental procedures were certified and approved by the Institutional Animal Care and Use Committee (IACUC) of Yonsei University Health System, which is certified by the Association for Assessment and Accreditation of Laboratory Animal Care International (AAALAC). All experimental procedures were conducted according to the guide for the care and use of laboratory animals (8th edition) by the National Research Council Committee, USA. All mice were housed under controlled environment with 12-h light/dark cycles and temperature, and ad libitum access to chews and water in a specific pathogen-free (SPF) facility at the Yonsei Biomedical Research Institute. The present experiment was performed according to an approved animal protocol (No. 2016-0335). The study is reported in accordance with the ARRIVE guidelines for reporting experiments involving animals (http://www.nc3rs.org.uk/arrive-guidelines).

### Experimental design and procedure

**Figure 1:**
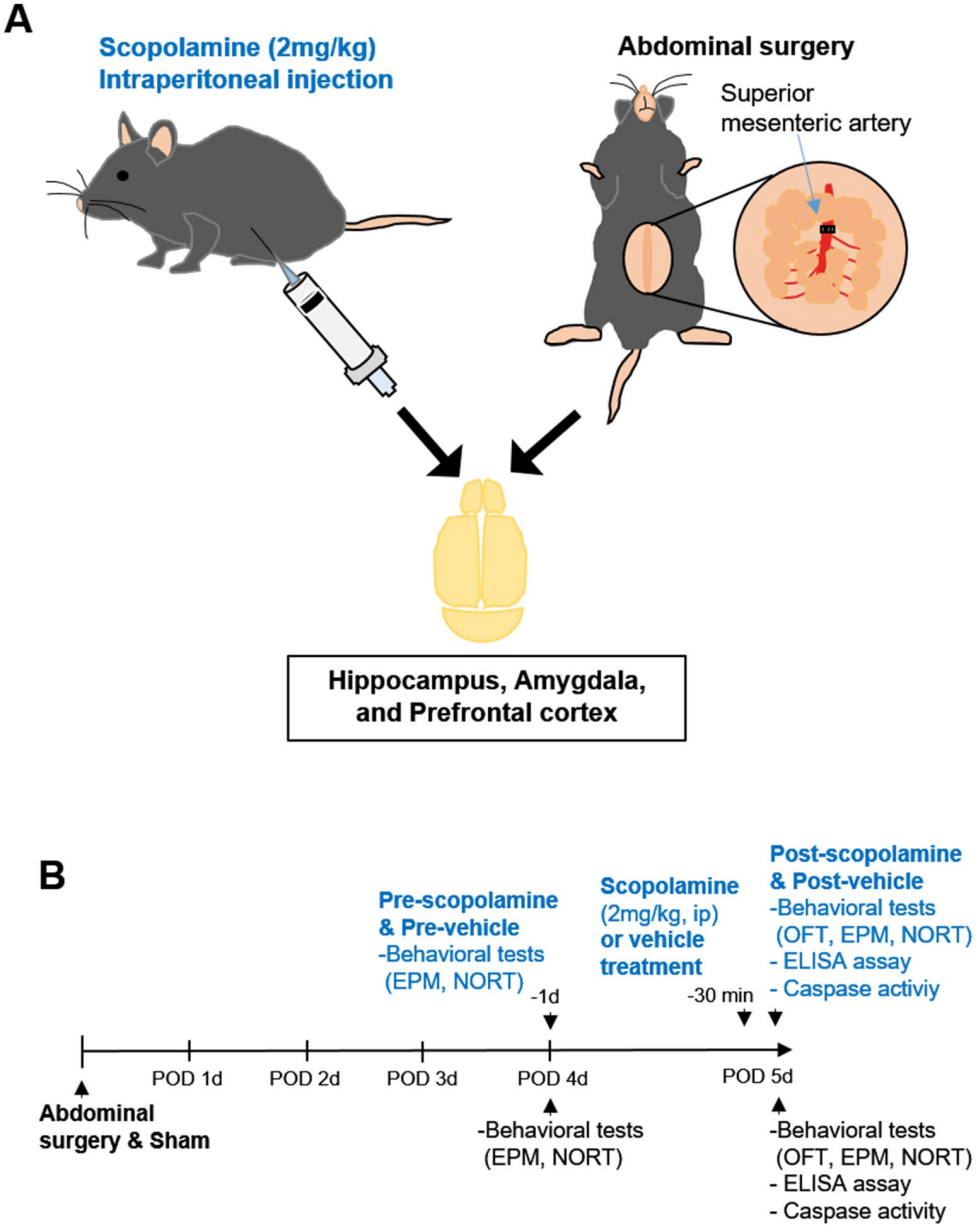
Experimental design and procedure. (**A**) Scopolamine (2 mg/kg) or vehicle was injected intraperitoneally. Mice were subjected to abdominal surgery by clipping superior mesenteric artery and rubbing intestines. All mouse brains were isolated, and brain sub-regions, such as hippocampus, prefrontal cortex, and amygdala, were analyzed after surgery or scopolamine treatment. (**B**) Mice in surgery group and sham group performed behavioral test at 4 and 5 day post-surgery. Mice in both vehicle and scopolamine-treated groups performed behavioral test at 1 day before scopolamine or vehicle treatment, and 30 min after scopolamine or vehicle treatment.

The mice were assigned into four groups using appropriate randomization methods: (1) a sham group (sham) (n = 10, male), (2) a surgery group (surgery) (n = 10, male), (3) a vehicle-treated control group (vehicle) (n = 10, male), and (4) a scopolamine-treated group (scopolamine) (n = 10, male). Mice in the sham group were kept unaffected to the experimental conditions, while mice in the surgery group underwent abdominal surgery. For the scopolamine-treated group, scopolamine (2 mg/kg)^21,22^ was dissolved in sterile saline (0.9% NaCl w/v) with the volumes for the administration prepared according to the body weight. Mice in the surgery and sham group performed behavioral test at day 4 and 5 postsurgery. After behavioral test, mice were sacrificed. Mice in both the vehicle and scopolamine-treated groups performed behavioral test at day 1 before scopolamine or vehicle treatment (4th day of the sham and surgery group). At day 2 (5th day of the sham and surgery group), the mice were injected with scopolamine intraperitoneally 30 minutes (min) before the neurobehavioral tests. The same amount of sterile saline was administrated into mice of the vehicle group at 30 min before the neurobehavioral tests. After neurobehavioral tests, vehicle or scopolamine injected mice were sacrificed at 3 h after injection (Fig. 1A,B). The surgical procedure was as follows. In the surgery group, mice were anesthetized with 4% isoflurane and were maintained with 1.5–2% isoflurane in oxygen at a flow rate of 1 L/min. Mice were placed on a heating pad during anesthesia to prevent hypothermia. As abdominal surgery is well-established interventional method in developing POCD model^23,24^, it was performed in surgery group mice as mentioned previously with some modifications in this study^25,26^. After incision of the peritoneum, superior mesenteric artery was separated and clipped for 20 min to mimic the abdominal surgery in mice. Concomitantly, small intestines were exposed and rubbed for 30 seconds (sec) by the operator to mimic the actual clinical surgical procedures. The small intestines and exteriorized abdominal muscle and skin were placed back into the peritoneal cavity and closed using sutures. Tramadol (20 mg/kg) was administrated intraperitoneally to mice undergoing surgery for postoperative analgesia. In sham group, mice were anesthetized following the same method, however the surgical procedure was omitted in this group. Mice were returned to the home cage. At day 5 postsurgery or 3 h after scopolamine treatment, mice brains, including the hippocampus, prefrontal cortex, and amygdala, were isolated after sacrifice.

### Neurobehavioral assessment

The neurobehavioral findings of the mice were assessed using the open field test (OFT), elevated plus maze (EPM) and novel object recognition test (NORT). The behavioral tests were performed 1 day before drug administration or 4 days after surgery. Mice underwent additional follow-up behavioral test 30 min after drug administration or 5 days after surgery according to the treatment plan. All the neurobehavioral tests were automatically video recorded and analyzed with an image analyzing system (SMART v2.5.21 software and SMART video tracking system, Panlab Harvard Apparatus, Barcelona, Spain) by an assessor blinded to the treatment groups. The mice sequentially performed the OFT, EPM and NORT.

### Open field test

Mice were placed in a square open field arena (40 × 40 × 40 cm), were allowed to explore for 5 min, and the behaviors were recorded simultaneously. Total distance moved was used as a measure of general activity and locomotor function. The animal’s tendency to avoid the center area reflects the anxiety related behavioral change^27^.

### Elevated plus maze

The EPM was performed to evaluate anxiety related behavior, learning, and memory functions of the mice^28–30^. The maze consisted of two open arms (31 × 6 × 1 cm) and two closed arms (31 × 6 × 15 cm) extended from a central platform (5 × 5 × 1 cm), and was elevated to a height of 50 cm (JEUNGDO Bio & Plant Co., Ltd.) from the floor. The mice underwent baseline experiments prior to surgery or scopolamine treatment, and tested again at day 5 postsurgery or 30 min after scopolamine treatment. Mice were individually placed at the end of the open arm facing the other open arm and were allowed to explore for 5 min. The total duration of time spent in the open and closed arms was recorded respectively. The percentage of time spent in the open arms was also measured. The duration of time spent in the open arms also reflects anxiety related behavior^30^. The apparatus was cleaned with 70% ethanol prior to all tests. Entry was defined as the placement of all paws into the arms of the maze. The ability of learning and memory was calculated as follows: Transfer latency = the first latency time to enter the closed arms (baseline/training period) – the first latency time to enter the closed arms (test period). Transfer latency reflects the changes in learning and memory function^28,29^.

### Novel object recognition test

NORT was performed to evaluate cognition, especially recognition memory in the mice^31^. During the habituation phase, each mouse was allowed to explore the square box (40 × 40 × 40 cm) freely for 5 min. During the familiarization phase, the mice were placed into the box, which contained two identical objects (A + A), and were allowed to explore for 5 min. During the test phase, each mouse was returned to the box with the two objects, where one object was changed into a novel object (A + B), and mice were allowed to explore for 5 min. During both the familiarization and the test phases, the time spent (%) in close-proximity exploration of each object was measured and recorded automatically. At the end of each test, the apparatus and the objects were cleaned with 70% ethanol. The habituation phase was performed immediately prior to surgery, the familiarization phase was performed at day 4 postsurgery, and the test trial was performed at day 5 postsurgery. The discrimination index was evaluated as: (time taken to explore novel object (B)) / (time taken to explore familiar and novel objects (A + B)) × 100, to reflect cognitive ability.

### Enzyme-linked immunosorbent assay (ELISA)

For the in vivo cytokine experiment, the hippocampus, prefrontal cortex, and amygdala of mice were obtained at day 5 postsurgery, or at 3 h after scopolamine or vehicle treatment after the neurobehavioral tests. Samples were stored at − 80 °C until use. To measure the levels of TNF-α, IL-1β, and IL-18 in the three different regions, brains were lysed using tissue protein extraction reagent (Tissue Protein Extraction Reagent, Thermo Scientific, Waltham, MA, USA) containing protease and phosphatase inhibitor cocktail (100 × Halt protease and phosphatase inhibitor cocktail, #1861281 Thermo Scientific). The tissues were then homogenized and centrifuged at 13,000 rpm for 10 min to obtain sample supernatants. Supernatant protein concentrations were measured with a BCA Protein Assay Kit (Thermo Scientific) according to the manufacturer’s specifications. Levels of TNF-α, IL-1β, and IL-18 in the lysates were assayed using high-sensitivity ELISA kits (R&D Systems Inc., Minneapolis, MN, USA) according to the manufacturer’s specifications. Briefly, samples were added to the assay plates at a volume of 50 μL/well and incubated for 2 h at room temperature. After washing plates with the wash buffer from the kit, TNF-α, IL-1β, and IL-18 conjugates were added to each well and incubated for 2 h. The absorbance of each well was measured at 450 nm using a microplate reader. To measure the levels of NLR family pyrin domain-containing protein 3 (NLRP3), apoptosis-associated speck-like protein containing a C-terminal caspase recruitment domain (ASC), and caspase-1 in lysates, ELISA kits from MyBioSource (San Diego, CA, USA) were used for this assay, and all procedures followed manufacture’s instruction.

### Caspase-1 activity assay

For measurement of caspase-1 activity, caspase-1 assay kit (Fluorometric) (Abcam, Cambridge, UK) was used. It was adopted for detecting caspase-1 activity, which recognizes the sequence of YVAD. The hippocampus, prefrontal cortex, and amygdala of mice were obtained at day 5 postsurgery, or at 3 h after scopolamine or vehicle treatment. According to manufacturer’s protocol, prepared samples were added to 96 well-plate. Then, reaction buffer and YVAD-AFC substrate were added and incubated for 1 h at 36 °C. After the reaction, the fluorescence (Ex/Me = 400/505 nm) of each well was measured using FlexStation Multi-mode microplate reader.

### RNA extraction and gene expression profiling

At day 5 postsurgery or 3 h after scopolamine treatment, mice brains were separated after sacrifice. Total RNA from mouse hippocampus tissue was extracted using Trizol reagent (Invitrogen, Carlsbad, CA, USA). RNA quality and quantity were assessed using Agilent 2100 bioanalyser (Agilent Technologies, USA) and ND-1000 spectrophotometer (NanoDrop Technologies, USA), respectively. RNA samples were used as input into the Affymetrix procedure (Affymetrix, Santa Clara, CA, USA) as recommended by protocol (http://www.affymetrix.com), of which total RNA from each sample was converted to double-strand cDNA. Amplified RNA (cRNA) was generated from the double-stranded cDNA template through an IVT (in vitro transcription) reaction using a random hexamer incorporating a T7 promoter and purified with the Affymetrix sample cleanup module. cDNA was regenerated from a random-primed reverse transcription using a dNTP mix containing dUTP. UDG and APE 1 restriction endonucleases were used for fragmenting cDNA, which was then end-labelled by terminal transferase reaction incorporating a biotinylated dideoxynucleotide. Fragmented end-labeled cDNA was hybridized to the Affymetrix arrays for 16 h (45 ℃ and 60 rpm) as described in the Gene Chip Whole Transcript (WT) Sense Target Labeling Assay Manual (Affymetrix). The chips were stained using SAPE (Streptavidin Phycoerythrin), washed in a Genechip Fluidics Station 450 (Affymetrix) and scanned using Affymetrix Model 3000 7G scanner. The scanned image data were extracted through Affymetrix Command Console 1.1 software to generate raw CEL files, which show expression intensity data. Expression data were generated by Transcriptome Analysis Console 4.0.1. For the normalization, RMA (Robust Multi-Average) algorithm implemented in Transcriptome Analysis Console software was used.

### RNA sequencing analysis of differentially expressed genes

Genes with a more than two-fold difference in the normalized signals compared to those in control group were selected as differentially expressed genes (DEG). Gene ontology analysis of the DEGs was performed by exDEGA (Excel based Differentially Expressed Gene Analysis, eBIOGEN, Inc., Seoul, Korea) tool. Categorization of the genes was based on a search performed using DAVID v6.8 (http://david.abcc.ncifcrf.gov). In each group, gene expression level was converted to a log2 value, and the relative level with respect to the control group was presented. The clustering heatmap profiles of DEGs were compared across the experimental groups using the Multiple Experiment Viewer software program v4.9 (MeV). The average fold change (FC) for each gene was expressed as a standardized z-score.

### Statistical analysis

Statistical analyses were performed using GraphPad Prism 7.00 software (GraphPad Software, San Diego, CA, USA). Values are presented as mean ± standard error of the mean (SEM). Unpaired *t*-tests were performed to determine statistical significance in behavioral test and caspasae-1 activity. Statistical comparisons among groups in ELISA and RNA sequencing were assessed with a one-way ANOVA. *p* values < 0.05 were considered significant. All graphs are created by using GraphPad Prism 7.00 software (GraphPad software) and Fig. 1 image is creased by using Microsoft PowerPoint.

## Results

### Scopolamine treatment induces delirium-like cognitive dysfunction in mice

**Figure 2:**
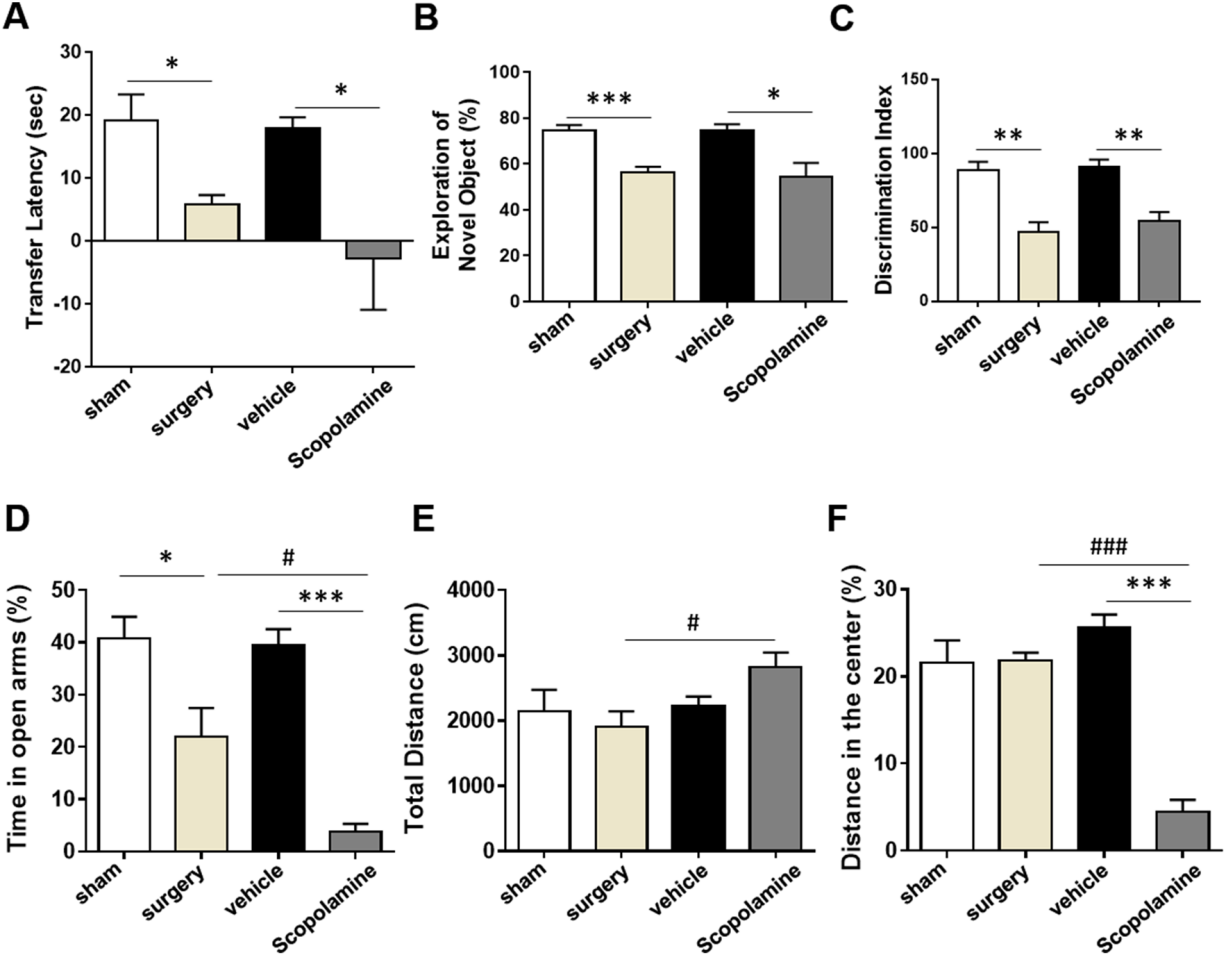
Effects of scopolamine on memory dysfunction and psychological behaviors in post-behavioral tests. (**A**) Changes in learning and memory is presented as the transfer latency (sec) in EPM, (**B**) the exploration time of novel object in NORT after surgery or scopolamine treatment, and (**C**) discrimination index in NORT after surgery or scopolamine treatment. (**D**) Reduced percentage time in open arms after surgery or scopolamine treatment in EPM. (**E**) Increased total distance travelled in OFT after surgery or scopolamine treatment. (**F**) Lower distance travelled in center zone of OFT after surgery or scopolamine treatment. Values are presented as mean ± SEM (n = 4–5). *p *values < 0.05 were considered significant (*p < 0.05, **p < 0.01, ***p < 0.001 vs. control (sham or vehicle), *#p* < 0.05, *##p* < 0.01, *###p* < 0.001 vs. surgery). EPM; elevated plus maze, NORT; novel object recognition test, EPM; elevated plus maze, OFT; open field test.

Delirium is related to accelerated cognitive dysfunction^2^. Therefore, we examined the role and validity of scopolamine treatment as the POD model by causing cognitive impairment, similar to POCD caused by the abdominal surgery model. Behavioral tests of EPM and NORT were carried out to assess the changes in learning and memory of mice after surgery or scopolamine treatment (Fig. 2A–C). In post-behavioral test, during the EPM test, surgery mice exhibited significant decrease in transfer latency (sec) as compared to sham group, which indicates POCD. Similarly, scopolamine-treated mice displayed a significant drop in transfer latency with no difference between the surgery and scopolamine groups (Fig. 2A), as compared to mice from the vehicle group. To examine the changes in learning and memory of mice after surgery or scopolamine treatment, post-behavioral tests were perform using the NORT to confirm the changes in learning and memory. The duration of time spent exploring the novel object (calculated in percentage using the given formula) significantly decreased in the surgery and scopolamine-treated groups, emphasizing the effect of surgery and scopolamine treatment on the impairment of cognitive ability (Fig. 2B). The discrimination index also showed reduced learning and memory ability in surgery or scopolamine treatment compared to each control (Fig. 2C). The SMART video tracking system showed real-time tracking of each groups in NORT (Supplementary Fig. 1A). Conclusively, neurobehavioral tests indicated that scopolamine treatment significantly caused memory impairments and decrease in cognition in mice, similar to the results seen in mice of the surgery group.

### Scopolamine treatment promotes delirium-like anxiety and hyper-activation in mice

We measured the level of anxiety to investigate whether scopolamine administration could lead to psychological disturbance, a characteristic trait of delirium. Mice behavior tests were conducted using EPM and OFT (Fig. 2D–F). In post-behavioral test, mice exhibiting the state of anxiety are known to display a tendency of staying in the closed arms of the EPM, thus, scopolamine-treated mice spent significantly less amount of time in the open arms as compared to mice from the vehicle and surgery groups, and surgery mice also showed less time in open arms, as compared to those in the sham group (Fig. 2D). In post-behavioral test, in OFT, there was no significant difference between the total distance travelled by the sham and surgery group mice, However, mice in the scopolamine-treated group exhibited hyperactive motor activity resulting in significantly greater total distance travelled, as compared to mice in the surgery group (Fig. 2E). Additionally, scopolamine-treated mice showed lesser distance travelled in the central zone of the OFT (Fig. 2F), suggesting the increased level of anxiety. The SMART video tracking system showed visual tracking of each group in EPM and OFT (Supplementary Fig. 1B and C).

### Transcriptome analysis shows scopolamine treatment alters gene expression pattern in the hippocampus

**Figure 3:**
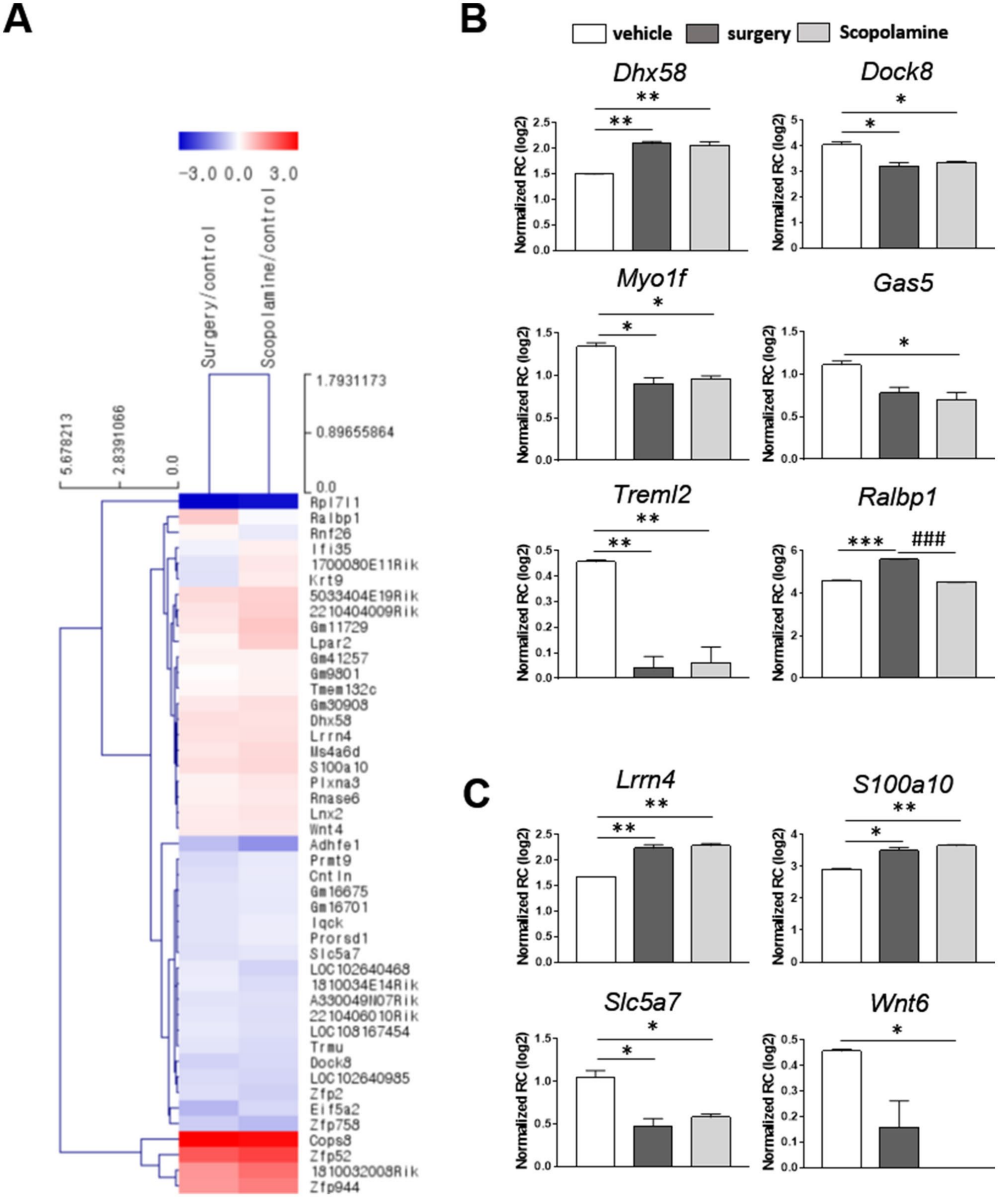
RNA sequencing transcriptome analysis. (**A**) Hierarchical clustering of hippocampal samples from vehicle, surgery, and scopolamine-treated groups. Samples were clustered according to the gene expression profiles of differentially expressed genes between control, surgery, and scopolamine-treated mice, respectively. The clustering tree is shown on the left, and the sample clustering tree appears at the top. The color scale shown at the top indicates the relative gene expression levels, with red representing a high expression level and blue, a low expression level. (**B**) Differentially expressed mRNAs involving immune/inflammatory response, and (**C**) hippocampal functionality and nervous system after surgery or scopolamine treatment. Values are presented as mean ± SEM (n = 2–3). *p *values < 0.05 were considered significant (*p < 0.05, **p < 0.01, ***p < 0.001 vs control (sham or vehicle), *###p* < 0.001 vs surgery).

To investigate whether surgery or scopolamine treatment affects the gene expression pattern in animal models, we used RNA sequencing analysis to compare the gene expression of hippocampal samples from the vehicle, surgery, and scopolamine-treated mice. The heat map showed the two-way hierarchical clustering comparing the gene expression levels among different groups: surgery or scopolamine (Fig. 3A). *Dock8*, *Myo1f.*, *Treml2*, *Gas5*, and *Ralbp1*, were upregulated or downregulated after surgery or scopolamine treatment. The normalized repeatability coefficient (RC) of *Dhx58*, which is involved in process of the immune system, was upregulated in both the surgery and scopolamine-treated groups, as compared to the control group. The gene expression levels of *Dock8*, *Myo1f.*, and *Treml2* were downregulated in both the surgery and scopolamine-treated groups. In the case of *Gas5*, only the surgery group showed downregulated level, as compared to the control. Compared to the surgery group, the level of *Ralbp1* was downregulated in both the vehicle and scopolamine-treated groups (Fig. 3B). In addition, genes involving hippocampal function and the nervous system, such as *Lrrn4*, *S100a10*, *Slc5a7*, and *Wnt6* were altered by surgery or scopolamine treatment. Gene expression of *Lrrn4* and *S100a10* was upregulated, while that of *Slc5a7* was downregulated in both the surgery and scopolamine-treated groups, as compared to the vehicle group. However, only the scopolamine-treated group showed efficiently downregulated expression of *Wnt6*, as compared to the vehicle and surgery groups (Fig. 3C).

### Scopolamine treatment increases pro-inflammatory cytokines and inflammasome components in the brain

**Figure 4:**
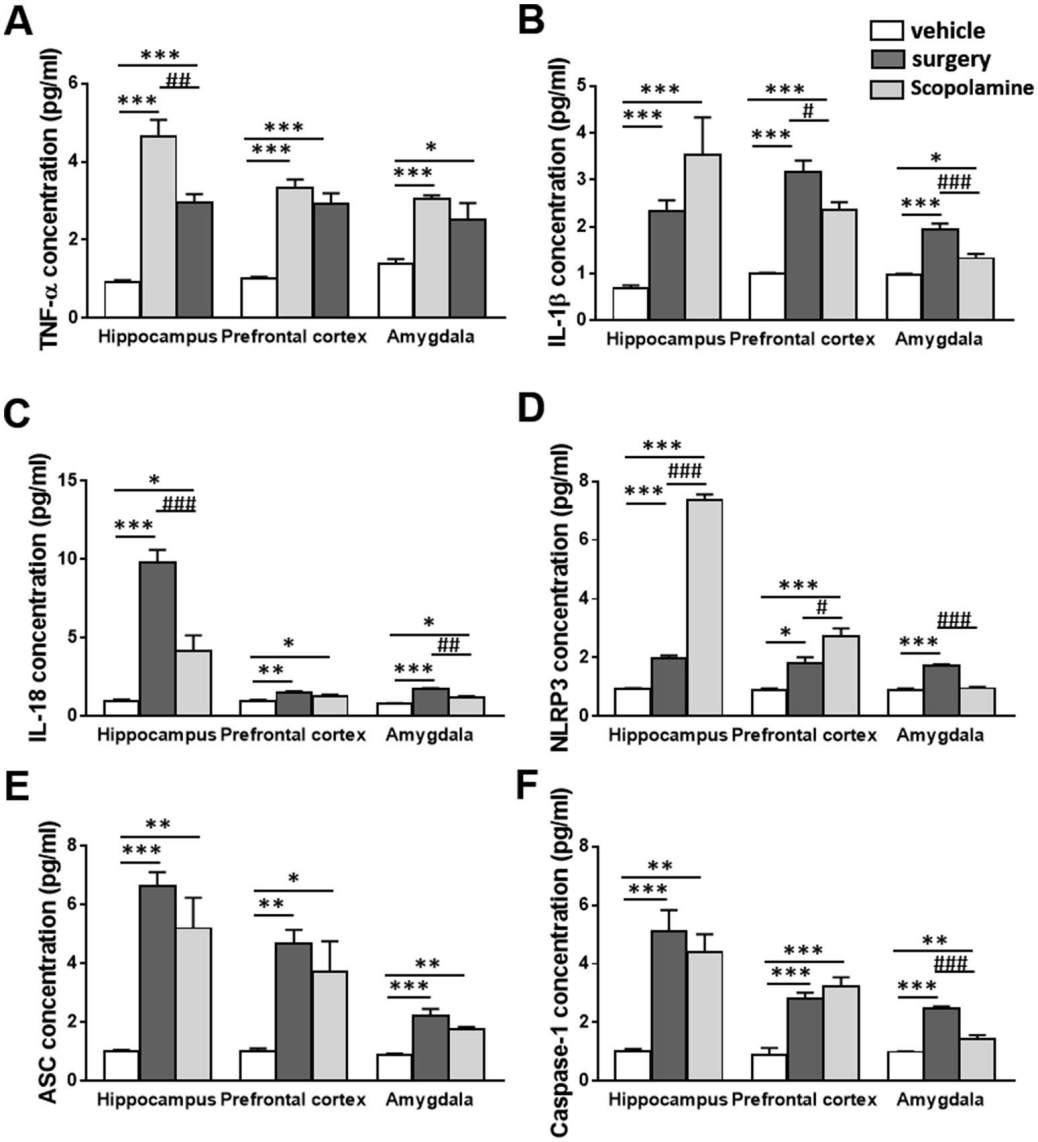
The levels of pro-inflammatory cytokines and NLRP3 inflammasome components in the brain regions. (**A**–**C**) Increased levels of pro-inflammatory cytokines including TNF-α, IL-1β, and IL-18 in the hippocampus, prefrontal cortex and amygdala with surgery or scopolamine treatment. (**D**–**F**) Upregulated levels of NLRP3, ASC, ad caspase-1 in the hippocampus and prefrontal cortex with surgery or scopolamine treatment. Also, increased levels of ASC, ad caspase-1 in the amygdala with surgery or scopolamine treatment except NLRP3. Values are presented as mean ± SEM (n = 4–6). *p* values < 0.05 were considered significant (*p < 0.05, **p < 0.01, ***p < 0.001 vs control (sham or vehicle), *#p* < 0.05, *##p* < 0.01, *###p* < 0.001 vs surgery).

Based on the changes of inflammatory genes in RNA sequencing analysis, the levels of pro-inflammatory cytokines were measured from the hippocampal samples using ELISA to compare the effects of surgery (day 5 postsurgery) and scopolamine (3 h after injection) on the inflammatory reaction (Fig. 4). Both surgery and scopolamine-treated mice showed significantly increased level of pro-inflammatory cytokines (TNF-α, IL-1β, and IL-18) (Fig. 4A–C). The levels of pro-inflammatory cytokines IL-1β and IL-18 are regulated by inflammasome. Therefore, we measured protein levels of inflammasome components in the hippocampus. The expression level of NLRP3 inflammasome components such as NLRP3, ASC, and caspase-1 in the hippocampus were significantly upregulated in both the surgery and scopolamine-treated groups, as compared to the vehicle group (Fig. 4D–F). The prefrontal cortex and amygdala are engaged in emotional response and mood regulation, such as anxiety and depression^32^. We further checked the levels of pro-inflammatory cytokines and NLRP3 inflammasome components in the prefrontal cortex and amygdala. Compared to the vehicle group, levels of pro-inflammatory cytokines, such as TNF-α, IL-1β, and IL-18, were remarkably higher in the surgery group. Additionally, mice of the scopolamine-treated group showed increased levels of TNF-α, IL-1β, and IL-18; although, IL-1β and IL-18 protein levels in scopolamine-treated mice were significantly reduced as compared to those in the surgery group (Fig. 4A–C). The levels of NLRP3 inflammasome components, such as NLRP3, ASC, and caspase-1, were highly expressed in the prefrontal cortex and amygdala in the surgery group. Similar to the surgery group, the scopolamine-treated group showed increased levels of NLRP3 inflammasome components in the prefronal cortex. Furthermore, the levels of NLRP3 in the amygdala were different between the mice in the surgery and scopolamine-treated groups with no incremental changes in the latter. However, ASC and caspase-1 expressions were higher in the scopolamine-treated group (Fig. 4D–F).

### Scopolamine treatment induces inflammasome complex-induced caspase-1 activation

**Figure 5:**
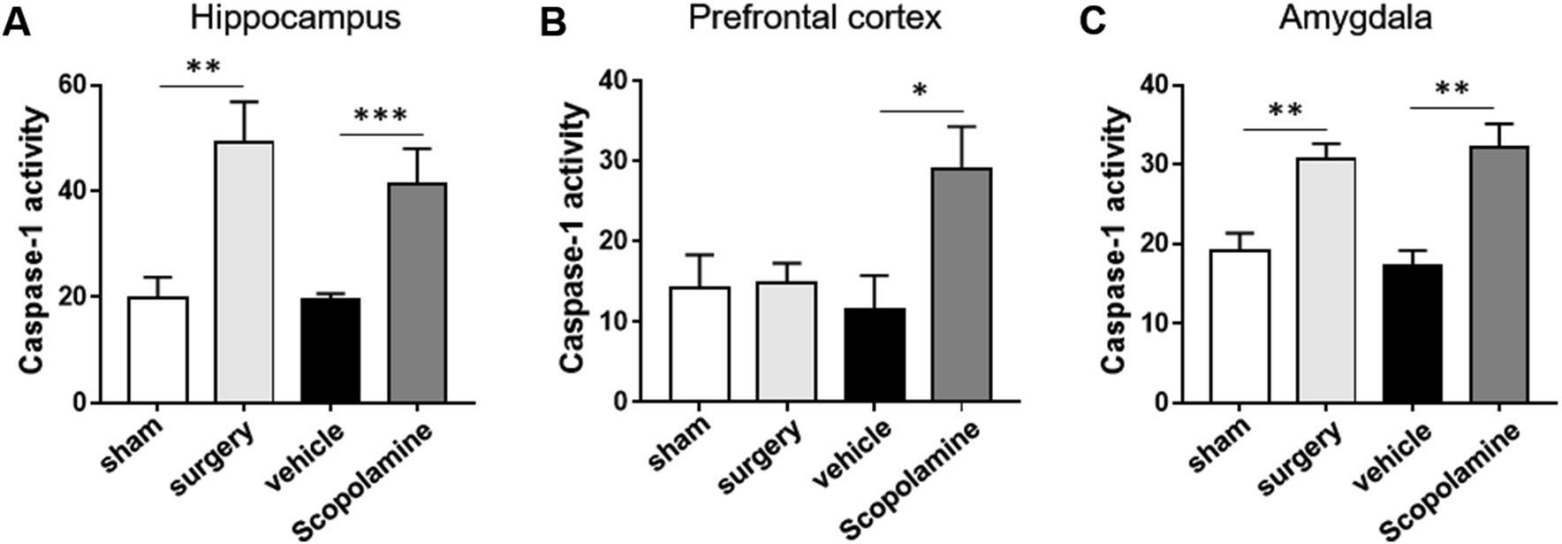
The levels of caspase-1 activity in the brain regions. (**A**) Highly activated level of caspase-1 after surgery (day 5 postsurgery) and scopolamine (3 h after injection) in the hippocampus. (**B**) Increased caspase-1 activation in the prefrontal cortex after scopolamine treatment. (**C**) Increased levels of caspase-1 activity in the amygdala in surgery and scopolamine groups. Values are presented as mean ± SEM (n = 5). *p *values < 0.05 were considered significant (*p < 0.05, **p < 0.01, ***p < 0.001 vs. control (sham or vehicle)).

The degree of caspase-1 activity was measured in the hippocampus, prefrontal cortex, and amygdala at day 5 postsurgery or 3 h after scopolamine injection (Fig. 5). In Fig. 5A, surgery and scopolamine group showed increased levels of caspase-1 activation, compared to each control in the hippocampus. In the prefrontal cortex, only scopolamine group showed increased caspase-1 activity, compared to the vehicle group. However, no significant differences were seen between the sham and surgery groups (Fig. 5B). In the amygdala, mice in surgery group displayed increased caspase-1 activity, compared to the sham group, while scopolamine-treated mice also showed increased levels of caspase-1 activation, compared to the vehicle group (Fig. 5C).

## Discussion

In the present study, scopolamine-treated animals showed characteristic delirium-like behavioral patterns, biochemical findings coherent with neuroinflammatory changes, and the activation of inflammasome. RNA sequencing profiles also showed consistent changes in gene expression patterns relevant to immune/inflammatory reaction and the nervous system development in both surgery and scopolamine-treated groups, though there are some discrepancies in the expression patterns of inflammation-related genes. These results suggest the compatibility of scopolamine-treated delirium-like animal model in re-enacting the clinical features of POD, although there could still be undisclosed pathophysiology and insufficient explanation for neuroinflammatory-related POD.

The cholinergic neurotransmitter system is responsible for the control of cognitive processes, acquisition, and retention of information as well as task performances. Numerous studies have reported the role of cholinergic function in age-related memory dysfunction and other neurocognitive diseases^33,34^. Scopolamine is known to reproduce delirium-like state in both human and experimental animals^35–37^ by inducing dysregulation of cholinergic signals in the brain; such cognitive declining properties of scopolamine have contributed to its wide application in neurocognitive research.

Oxidative stress has also been proposed as the possible cause for neurocognitive disease along with the cholinergic hypothesis. Regarding to the use of scopolamine, previous studies have reported the association of systemic administration of scopolamine with increased oxidative stress in the brain, especially the areas associated with memory and learning, such as the hippocampus and prefrontal cortex^16,38^. Although dysregulation of the cholinergic system and oxidative stress have been the promising hypothesis for explaining the pathogenesis of POCD and delirium, increased inflammation still plays an important part in explaining the development of neurocognitive disorders, of which increased level of pro-inflammatory cytokines, such as TNF-α IL-1β, IL-18, and IL-6, can be observed in the brain and blood samples of patients with dementia, delirium, and POCD^39–41^. Scopolamine administration has also been shown to cause increase in pro-inflammatory cytokines, and inflammation has been proposed as the theoretical basis for scopolamine-induced memory impairment^16,42^. The significance of neuroinflammation in delirium is consistent with our results in showing the increase of pro-inflammatory cytokines in both the surgery and scopolamine-treated groups.

NLRP3 inflammasome, an intracellular sensor that detects a broad range of microbial motifs, has been demonstrated to increase the level of pro-inflammatory cytokines such as IL-1β and IL-18 in the brain by activating caspase-1^43^. NLRP3 inflammasome further promotes the aggregation of innate immune cells and initiates the downstream inflammatory cascade that ultimately accelerates the pathological progression of neurocognitive disease^43–45^. Its activation had been the marker for host immune defense mechanism in various inflammatory diseases with no exceptions to the development of neurodegenerative disorders. In this study, the activation of NLRP3 inflammasome and caspase-1 was observed in three different brain regions, of which the hippocampal area of scopolamine-treated mice showed increased activation. Increased activation of NLRP3 inflammasome was also seen in the hippocampus of surgery mice implying POCD. Therefore, NLRP3 inflammasome may be the main cause of cognitive impairment after surgery as well as scopolamine treatment.

Amygdala is the brain region crucial for executive function, memory, attention, and especially for the pathogenesis of delirium, along with the frontal lobes, diencephalon and hippocampus^37,46^. In addition, the dysregulated prefrontal cortex-to-amygdala pathway is associated with anxiety^32^. Previous studies have shown the significant diminution of neurotransmitters, including dopamine, 3,4-dihydroxy-phenylacetic acid (DOPAC), homovanillic acid (HVA), and acetylcholine, in the amygdala of scopolamine-treated animals as compared to control group animals, and also compared to the hippocampus area within the same scopolamine-treated groups^37,46^. In addition, evidences have also suggested altered neurotransmission especially in the regions related to memory, such as the hippocampus and amygdala, in relation to outcomes seen in POD. The significance of inflammasome is very important in the neuroinflammatory-based hypothesis for POD. In the present study, the levels of NLRP3 inflammasome components and caspase-1 activity were all higher in the prefrontal cortical area of scopolamine-treated mice; however, the levels of expression were different in the amygdala, showing increase in the levels of inflammasome components and caspase-1 activity after scopolamine treatment, but not that of NLRP3. Detailed explanation for transition aspects of NLRP3 inflammasome in the amygdala could be limited due to restricted information focusing on the direct changes of inflammasome components in the amygdala, of which reduced expression of NLRP3 inflammasome can only be indirectly inferred from altered or abnormal expression of neurotransmitters. Also, other kinds of inflammasome complexes (e.g. NLRP2, NLRC4, and AIM2) might be involved in caspase-1 activation and inflammatory responses in the amygdala. However, in present study, we performed ELISA test for inflammatory cytokines except sham group, because there were no differences between the vehicle and sham groups in post-behavioral tests.

RNA sequencing analysis technique has been widely used for various human disease, and a large amount of genetic data has been produced over the past decades from various diseases. With the help of RNA sequencing analysis from different treatment groups, this study successfully identified the altered gene expression patterns from each group to further investigate the reliability of scopolamine-treated animal models as compatible delirium-like model from neuroinflammatory point of view. We have selected hippocampus as it is the specific brain region critical for learning and memory process, and known to have significant association with the neuropsychiatric behavioral changes by various studies. Other previous studies had also extracted RNA from hippocampal tissue to examine the RNA changes in neurobehavioral insults and changes in cognition and memory^47,48^. After scopolamine treatment, mice showed decreased expression patterns of cholinergic genes (*Chrnb3*, *Chrnb4*, *Chat*, *Camk2b*, *Slc18a3*, *Chrm1*) compared to the vehicle, although statistical significances were lacking. However, scopolamine treatment significantly reduced the expression levels of cholinergic genes (*Chrna3*, *Slc5a7*, *Slc38a5*) compared to the vehicle. This study also identified the genes with significant expression profiles related to inflammation and the development of the nervous system that presented with a more distinct expression pattern in surgery mice as compared with scopolamine- treated mice (Table 1). For example, gene expression of *Ralbp1*, which was significantly upregulated in surgery mice compared to mice in sham group and downregulated in scopolamine-treated mice compared to surgery mice*,* plays a role in receptor-mediated endocytosis and is a downstream effector of the small GTP-binding protein RAL^49^. *Ralbp1* is known for its oncogenic role and necessary role in cell proliferation and invasion in carcinogenesis^50^, and it is also known for regulating obesity-promoting pro-inflammatory cytokines^51^. Gene expression patterns from both the surgery and scopolamine-treated groups showed that a number of notable genes were associated with the analogous cellular functions, mostly focusing on inflammatory reactions and the development of the neuronal system, although expression levels and significance vary to some extent. For example, *Dhx58* coding LGP2, is related to the regulation of innate immune response, immune system, and apoptosis during viral infection^52^. *Myo1f.* encodes myosin-1F protein and is expressed mainly in the immune system^53^. It is involved in the regulation of M1-polarization during the inflammatory process, whereas *Myo1f.* deficiency is known to attenuate the commitment of macrophages into a pro-inflammatory phenotype. *Myo1f.* deficiency model strongly reduces the secretion of pro-inflammatory cytokines, decreases epithelial damage, ameliorates disease activity, and enhances tissue repair^54,55^. *Treml2* is a transmembrane protein family, which expresses in various immune cells, such as monocytes, macrophage, and microglia. *Treml2* is involved in innate and adaptive immunity and causes increase in number of macrophages under inflammatory condition^56^.

**Table 1:** Differentially expressed genes in surgery and scopolamine-treated mice.

	Surgery		Scopolamine		Gene description	GO biological process (gene function)
	FC	*p* value	FC	*p* value		
*Inflammation/immune response*						
*Dhx58*	1.516	0.003	1.488	0.020	DEXH (Asp-Glu-His) box polypeptide 58	Immune system process Regulation of Innate immune response
*Dock8*	0.559	0.016	0.612	0.044	Dedicator of cytokinesis 8 Inflammation	Protein binding Immunological synapse formation
*Myo1f.*	0.740	0.017	0.767	0.023	Myosin 1F Regulator of anti-inflammation	ATP, acting binding Defense response to Gram(+) bacterium
*Gas5*	0.797	0.032	0.754	0.045	Growth arrest specific 5 Molecular function Response to bacterium	
*Treml2*	0.752	0.003	0.762	0.019	Triggering receptor expressed on myeloid cells-like 2 Regulation of innate immune response	
*Ralbp1*	1.999	< 0.001	0.945	0.024	ralA binding protein 1 Promotion of pro-inflammatory cytokine (in obesity)	ATPase activity/protein binding Positive regulation of GTPase activity Signal transduction
*Hippocampal functionality/nervous system*						
*Lrrn4*	1.482	0.010	1.526	0.008	Leucine rich repeat neuronal 4 Hippocampus-dependent long-lasting memory	Molecular function Long-term memory
*S100a10*	1.518	0.028	1.677	0.004	S100 calcium binding protein A10 (calpactin) Hippocampus	Ion channel binding Protein binding Positive regulation of GTPase activity
*Slc5a7*	0.675	0.017	0.723	0.045	Solute carrier family 5 (choline transporter), member 7 Hippocampus	Choline binding/choline transmembrane transporter activity Acetylcholine biosynthetic process Choline transport
*Wnt6*	0.818	0.098	0.729	< 0.001	Wingless-type MMTV integration site family, member 6	Protein/signaling receptor binding Neuron differentiation Positive regulation of gene expression

RNA sequencing data show several genes related to inflammation and the development of the nervous system in surgery mice and scopolamine- treated mice.

Another gene, *Lrrn4*, which was upregulated in both the surgery and scopolamine-treated groups, is a protein coding gene playing a crucial role in hippocampus-dependent long lasting memory^57^. *S100a10* encodes a member of the S100 family of protein containing 2 EF-hand calcium-binding motifs called S100A10 or p11, which are involved in the regulation of various cellular processes such as cell cycle progression and differentiation. Upregulated levels of *S100a10* in the hippocampus engage in processing emotional memory and altered hippocampal functionality^58^, which is due to the interaction of p11 with serotonin-signaling proteins. Moreover, as cognitive impairments are common in delirium due to dysfunctional serotonin neurotransmission, p11 protein is known to interact with serotonin-signaling proteins and correlate with symptoms of mood disorders^58,59^. *Scl5a7* is implicated in the delivery of the precursor choline from the synaptic space into presynaptic terminal, which is important in cholinergic neuronal communication^60^. To summarize, scopolamine administration can successfully reproduce characteristic neuropsychiatric behavioral changes of delirium in accordance with neuroinflammatory hypothesis, although less consistency was noted in descriptive RNA sequencing analysis studies. In this study, we have only suggested candidate genes involved in scopolamine-treatment delirium. Therefore, further studies focusing on delirium and the involvement of candidate genes for pathophysiological studies are needed.

Delirium represents the large spectrum of cognitive and behavioral abnormalities from hypoactive form, with negative symptoms of inattention and flat affect, to hyperactive form with characteristic agitation and anxiousness^61^. In this study, neurobehavioral tests showed significant increase in the level of anxiety and hyper motor activity, and impairment in memory and cognitive function in scopolamine-treated group; this seems to be associated with increased level of inflammatory response. Scopolamine is particularly known to damage learning and short-term memory functions in rodents and humans by disrupting cholinergic transmission^16^, which makes it the most widely used drug for reproducing delirium in animal. Therefore, scopolamine treatment model has its advantage over other models (Especially a single IP injection of scopolamine) as suitable for POD study, while abdominal surgery in animal may be the proper and widely adopted method in developing POCD as supported by the previous studies^23,24^, but lacks the definite explanation for POD development.

In conclusion, our results show that scopolamine-induced POD animal models had succeeded in showing the analogous neurobehavioral patterns, and thus, present findings coherent with neuroinflammatory reactions in POD. However, genetic analysis showed the limitations as an indispensable explanation between candidate genes and delirium. Undeniably, further studies addressing the possible interactions between delirium and candidate genes after scopolamine treatment for pathophysiological studies are necessary.

## Supplementary Information

Supplementary Figure 1.

Supplementary Legend.

## Data Availability

All data supporting the conclusions of this manuscript are provided in the text and figures.
