# Supplementary figures and images for: Scopolamine promotes neuroinflammation and delirium-like neuropsychiatric disorder in mice
Source: Sci Rep. 2021 Apr 16;11:8376. doi: 10.1038/s41598-021-87790-y (PMC8052461; doi:10.1038/s41598-021-87790-y)

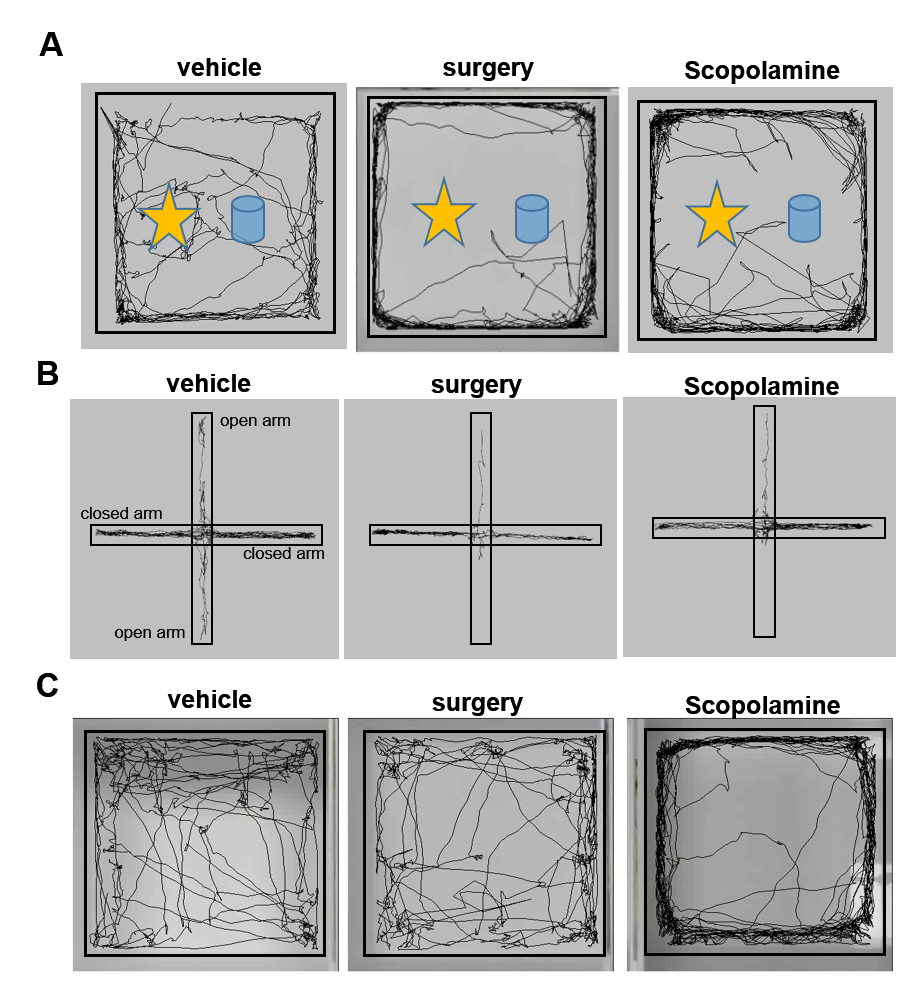

Supplement: Supplementary file 1 — Supplementary Figure 1. [file 41598_2021_87790_MOESM1_ESM.tif]
