# Supplementary material for: Scopolamine promotes neuroinflammation and delirium-like neuropsychiatric disorder in mice
Source: Sci Rep. 2021 Apr 16;11:8376. doi: 10.1038/s41598-021-87790-y (PMC8052461; doi:10.1038/s41598-021-87790-y)
Supplement: Supplementary file 2 — Supplementary Legend. [file 41598_2021_87790_MOESM2_ESM.docx]

**Supplementary Figure 1. Video tracking in post-behavioral tests** (A) Representative activity tracking during OFT. (B) Representative activity tracking during EPM. (C) Representative activity tracking during OFT.
